# Dynamics of plasma biomarkers in Down syndrome: the relative levels of Aβ42 decrease with age, whereas NT1 tau and NfL increase

**DOI:** 10.1186/s13195-020-00593-7

**Published:** 2020-03-19

**Authors:** David Mengel, Wen Liu, Robert J. Glynn, Dennis J. Selkoe, Andre Strydom, Florence Lai, H. Diana Rosas, Amy Torres, Vasiliki Patsiogiannis, Brian Skotko, Dominic M. Walsh

**Affiliations:** 1 Laboratory for Neurodegenerative Research, Ann Romney Center for Neurologic Diseases, Brigham and Women’s Hospital and Harvard Medical School, 60 Fenwood Road, Boston, MA 02115 USA; 2 Department of Neurodegenerative Diseases, Center for Neurology and Hertie Institute for Clinical Brain Research, University of Tübingen, Tübingen, Germany; 3 Division of Preventive Medicine, Brigham and Women’s Hospital, Boston, MA USA; 4 Department of Forensic and Neurodevelopmental Sciences, Institute of Psychiatry, Psychology and Neuroscience, King’s College London, London, UK; 5 Division of Psychiatry, University College London, London, UK; 6 Department of Neurology, Massachusetts General Hospital and McLean Hospital, and Harvard Medical School, Boston, MA USA; 7 Athinoula A. Martinos Center for Biomedical Imaging, Massachusetts General Hospital, Charlestown, MA USA; 8 Down Syndrome Program, Division of Medical Genetics and Metabolism, Department of Pediatrics, Massachusetts General Hospital, Boston, MA USA; 9 Department of Pediatrics, Harvard Medical School, Boston, MA USA

**Keywords:** Alzheimer’s disease, Amyloid, Down syndrome, Dementia, Neurofilament light, Simoa

## Abstract

**Background:**

Down syndrome (DS) is the most common genetic cause of Alzheimer’s disease (AD), but diagnosis of AD in DS is challenging due to the intellectual disability which accompanies DS. When disease-modifying agents for AD are approved, reliable biomarkers will be required to identify when and how long people with DS should undergo treatment. Three cardinal neuropathological features characterize AD, and AD in DS—Aβ amyloid plaques, tau neurofibrillary tangles, and neuronal loss. Here, we quantified plasma biomarkers of all 3 neuropathological features in a large cohort of people with DS aged from 3 months to 68 years. Our primary aims were (1) to assess changes in the selected plasma biomarkers in DS across age, and (2) to compare biomarkers measured in DS plasma versus age- and sex-matched controls.

**Methods:**

Using ultra-sensitive single molecule array (Simoa) assays, we measured 3 analytes (Aβ42, NfL, and tau) in plasmas of 100 individuals with DS and 100 age- and sex-matched controls. Tau was measured using an assay (NT1) which detects forms of tau containing at least residues 6–198. The stability of the 3 analytes was established using plasma from ten healthy volunteers collected at 6 intervals over a 5-day period.

**Results:**

High Aβ42 and NT1 tau and low NfL were observed in infants. Across all ages, Aβ42 levels were higher in DS than controls. Levels of Aβ42 decreased with age in both DS and controls, but this decrease was greater in DS than controls and became prominent in the third decade of life. NT1 tau fell in adolescents and young adults, but increased in older individuals with DS. NfL levels were low in infants, children, adolescents, and young adults, but thereafter increased in DS compared to controls.

**Conclusions:**

High levels of Aβ42 and tau in both young controls and DS suggest these proteins are produced by normal physiological processes, whereas the changes seen in later life are consistent with emergence of pathological alterations. These plasma biomarker results are in good agreement with prior neuropathology studies and indicate that the third and fourth decades (i.e., 20 to 40 years of age) of life are pivotal periods during which AD processes manifest in DS. Application of the assays used here to longitudinal studies of individuals with DS aged 20 to 50 years of age should further validate the use of these biomarkers, and in time may allow identification and monitoring of people with DS best suited for treatment with AD therapies.

## Introduction

Trisomy of chromosome 21, commonly known as Down syndrome (DS), is the most frequent genetic cause of lifelong intellectual disability and occurs in over 7 million people worldwide [1]. DS is also the most common genetic cause of early onset Alzheimer’s disease (AD) [2], and in prospective studies, the cumulative incidence for dementia is around 90% [3–5]. Given that the AD-associated *APP* gene is on chromosome 21, and that rare DS individuals who lack a third copy of *APP* do not develop AD [6], overexpression of APP and accumulation of the amyloid β-protein (Aβ) are considered the main drivers of AD in DS.

Early clinical diagnosis of AD in people with DS is complex and challenging because patients with DS have a pre-existing intellectual disability, the extent of which varies from person to person. Reliable AD-specific biomarkers would greatly assist early diagnosis of AD in DS, and could facilitate clinical care planning, and timely treatment with disease-modifying AD therapies which may soon become available [7, 8]. Measurement of Aβ and tau by their detection in CSF using immunoassays or visualization of deposited proteins using PET imaging can reliably detect AD in at-risk individuals [9–11]. However, PET imaging is expensive and its application outside of clinical research remains limited. Collection of CSF, although routine in certain countries, remains unpopular with patients, especially if required more than once. Lumbar puncture and PET scans are particularly challenging in a vulnerable population such as people with DS. In contrast, collection of blood would be significantly easier for individuals with DS, making blood-based biomarkers of AD in DS the preferred option.

Postmortem studies of brains from individuals with DS reveal that in general, diffuse amyloid plaques appear in the late teens, and tau pathology emerges after age 35 [12, 13]. Whether blood-based biomarkers of AD evolve in a similar temporal pattern is not yet clear. Prior studies of putative AD biomarkers examined restricted age groups [14–16], and earlier studies employed sub-optimal assays [17]. Most previous investigations on AD biomarkers in DS were limited to analysis of plasma Aβ. Now with the advent of reliable methods, it is possible to assess whether tau and neurofilament light (NfL) are also altered in DS. NfL, a scaffolding cytoskeleton protein, is elevated in plasma in many neurodegenerative conditions [18], and several NfL assays are available [19]. Measurement of tau in plasma has been less straightforward. This is because tau is molecularly heterogeneous [20–22] and is present in blood at only minute levels. We recently developed an ultra-sensitive immunoassay, which detects forms of tau captured by the mid-region antibody BT2 (to aa 194–198) and detected with the N-terminal antibody Tau12 (to aa 6–13). In several studies, we have found that measurement of tau using this NT1 assay effectively discriminates AD from controls [21] and is elevated in patients with mild cognitive impairment who subsequently developed AD [23]. Other assays which target distinct epitopes of tau have been reported, but these have shown less consistent differences between AD and controls [24–26].

Here, we measured Aβ42, NT1 tau, and NfL in plasma from individuals with DS and age-matched controls. Our study cohort covered a broad age range, including participants as young as 3 months and as old as 68 years. The study had 2 primary objectives: (1) to assess changes of plasma Aβ42, NT1 tau, and NfL in DS across age, and (2) to compare biomarkers measured in DS plasma versus age- and sex-matched controls. The usefulness of any biomarker is influenced by a myriad of factors, key among which are the stability of the analyte(s) over time, e.g., day-to-day variability, and whether or not it is affected by diurnal factors and requires pre-sampling fasting. Prior to analyzing precious specimens from our unique DS-control matched cohort, we examined the in vivo stability of analytes in the plasma of 10 healthy volunteers collected at 6 time points over a 5 day interval.

## Material and methods

### Participants

#### PRECISION study cohort

Blood was collected from ten healthy volunteers twice a day at 2 day intervals over a 5 day period. Participant characteristics are presented in Supplemental Table 1 (Additional file 1). On each day, blood was collected in the morning between 7 and 10 am and in the evening between 4 and 7 pm. The average time between blood draws on the same day was 8.4 ± 1.0 h. Participants had fasted for at least 10 h prior to morning blood draws (average fasting period 11.2 ± 1.0 h), but fasting was not requested for blood collection done in the evening. Blood was collected into 8 mL ethylenediaminetetraacetic acid (EDTA)-treated tubes by peripheral venipuncture with a 21G butterfly needle. Plasma was processed as quickly as possible, and the average time between venipuncture to plasma isolation was 19 min. Specimens were centrifuged at 2000×*g* for 10 min at 4 °C, and the supernatant was carefully removed and aliquoted into 500 μL lots in 1.5 mL Eppendorf protein lobind tubes and stored at − 80 °C. The study was conducted in accordance with the local clinical research regulations and approved by the Partners Institutional Review Board (Walsh, BWH2017P000259), and all participants gave written informed consent.

#### Down syndrome and control participants

Plasma samples were taken from 100 people with Down syndrome. Samples were obtained from three centers. The bulk of the samples (*n* = 82) were collected prospectively at the Massachusetts General Hospital Down Syndrome Program. Additional samples came from the Center for Neuroimaging of Aging and Neurodegenerative Disease of the Massachusetts General Hospital (*n* = 13) and the LonDownS Consortium at King’s College London (*n* = 5). Fasting was not required, and the time of day at which blood was collected was not prescribed.

##### Massachusetts General Hospital Down Syndrome Program

Participants with Down syndrome and/or their legal guardians were consented during outpatient visits at the Massachusetts General Hospital Down Syndrome Program. The protocol was approved by the Partners Human Research Committee. Blood was drawn from the antecubital vein with a 21G needle and collected into EDTA-treated tubes. Plasma was isolated by centrifugation at 2000×*g* at 4 °C for 10 min, and aliquots processed and stored as described for the PRECISION cohort.

##### Center for Neuroimaging of Aging and Neurodegenerative Disease of the Massachusetts General Hospital

Participants with DS were recruited from the MGH Down syndrome research database. Experimental procedures were explained, and signed informed consent/assent was obtained prior to participation. The protocol was approved by the Institutional Review Board (2018P000898). Blood collection and processing was performed as described for the PRECISION study cohort.

##### LonDownS Consortium

Participants with DS were also recruited as a part of the LonDownS Consortium’s cohort study of Alzheimer’s disease [27]. Ethical approval was obtained from the North West Wales Research Ethics Committee (13/WA/0194). Written informed consent was obtained if participants could consent for themselves; otherwise, a consultee was asked to approve the individual’s inclusion. Blood samples were collected in EDTA tubes and processed within 2 h. Plasma was prepared by centrifuging samples for 10 min at 2200×*g* at 4 °C; the supernatant was aliquoted and stored at − 80 °C.

##### Boston Children’s Hospital and Partners Biobank

Specimens from age- and sex-matched control subjects were obtained from the Precision Link Biobank at Boston Children’s Hospital (BCH) and the Biobank at Partners HealthCare in Boston (MA, USA). Controls had no history of diseases of the central nervous system. Specifically, there was no evidence of brain tumor, normal pressure hydrocephalus, stroke, severe brain trauma, brain surgery, epilepsy, encephalitis, or dementia. Included participants were free of acute infectious disease.

Precision Link Biobank participants (*n* = 67) were enrolled throughout the hospital, across diverse clinical settings. In-person informed consent was obtained from all participants enrolling in the Biobank and provides permission to (1) access electronic health record data for research, (2) collect and use residual specimens produced as by-products of routine care, and (3) share de-identified data and specimens outside of the institution. Participants provided a 4 mL blood sample for research use. Whole blood was collected in EDTA tubes, centrifuged at 2000×*g* for 10 min at room temperature, with plasma removed and aliquoted into 0.5 mL microcentrifuge tubes. Aliquots were stored at − 80 °C in the Biobank Core Lab facility until requested. The Precision Link Biobank initiative is approved by the BCH Institutional Review Board (P00000159).

Additional samples (*n* = 33) and health information were obtained from the Partners HealthCare Biobank, a biorepository of consented patient samples at Partners HealthCare. The Partners HealthCare Biobank is approved by the Institutional Review Board (2009P002312). Blood was collected, and plasma generated as described above.

### Single molecule array immunoassays

Levels of Aβ42, NT1 tau, and NfL were quantified using single molecule array (Simoa) assays. All assays were performed by the same operator and conducted on the same automated HD-1 analyzer (Quanterix, Billerica, MA). Consumables and reagents other than certain antibodies were obtained from Quanterix. The NT1 tau assay was developed in-house [21], and commercial kits were used to measure Aβ42 and NfL.

#### NT1

This is a 3-step assay capable of detecting all forms of tau which contain residues 6–198 [21]. BT2 (194-198, Thermo, Waltham, MA, USA) was conjugated onto paramagnetic beads at 2 mg/mL and used for capture. Biotinylated Tau12 (6-13, Merck Millipore, Darmstadt, Germany) was used for detection.

The optimal plasma dilution (1:4) to minimize matrix effects was determined previously [21]. Plasma samples were thawed on ice, centrifuged at 14,000×*g* for 4 min, and the upper 90% of the supernatant transferred to Eppendorf protein lobind tubes and then diluted 1:4 with Tau 2.0 sample diluent reagent (Quanterix, Billerica, MA). Samples, standards, and blanks were analyzed at least in duplicate.

The Lower limit of quantitation (LLoQ) defined as the lowest standard (i) with a signal higher than the average signal for the blank plus 9 SDs, and (ii) allowed a percent recovery ≥ 100 ± 20%. In 4 runs over 4 days, the LLoQ was 0.25 pg/mL. The average percent coefficient of variation (%CV) of all samples measured in the study was 13.8%. Assay characteristics including selectivity, dilution linearity, and spike and recovery were reported previously [21].

#### Aβ42 and NfL

The Simoa® Aβ42 Advantage (Quanterix, Billerica, MA, USA) and Simoa™ NF-light® Advantage (Quanterix, Billerica, MA, USA) kits were used according to the manufacturer’s instructions. Reagents from a single lot were used for analysis of all specimens from either the PRECISION study cohort or the Down syndrome study cohort. To determine the optimal dilution factor, plasma samples from 73 healthy donors were diluted 1:4 and 1:8 and analyzed for Aβ1-42 and NfL, and the highest dilution factor that allowed reliable quantification of samples was used. Thereafter, specimens were diluted 1:8 for Aβ42 and 1:4 for NfL. As with specimens for the NT1 assay, plasma was centrifuged at 14,000×*g* for 4 min; the upper 90% transferred to a new Eppendorf protein lobind tube and diluted with sample diluent provided in the kits. LLoQs for the Aβ1-42 and NfL assays were calculated as described for the NT1 assay and were 0.41 pg/mL and 0.47 pg/mL, respectively. The average %CV for all samples measured in the study was 5.5% for the Aβ1-42 and 7.5% for the NfL assay.

### Statistical analyses

Statistical analyses were carried out using GraphPad Prism, version 8 (LaJolla, CA, USA), and Stata, version 15.1 (Stata Corp., College Station, TX, USA). The effect of daytime (morning vs. evening blood draws) and different weekdays (day 1 vs. day 3 vs. day 5) on plasma biomarker levels was assessed using linear mixed models (“mixed”). Both variables were included as fixed affects, whereas subjects were used as random effects. To assess goodness of fit, we included both variables in a stepwise process and compared Akaike information criterion (AIC) of the respective models (“estat ic”). Tests on estimated coefficients were performed using the Wald test postestimation command (“test”). Differences of biomarker levels between DS and controls for specified age ranges were assessed using a paired *t* test (for normally distributed data) or a Wilcoxon signed-rank test (for non-normally distributed data). *p* values were adjusted for multiple testing by Bonferroni correction. Additionally, piecewise linear regression using hockey-stick regression was applied to model the effect of age on plasma biomarker levels in DS and controls (and differences between both groups) [28]. The slopes of both regression lines and the change-point where both regression lines meet were estimated using the “nl hockey” function. From specimens of DS and age-matched control participants, 10 out of 600 measurements (1.7%, 7 for the NT1 tau and 3 for the NfL assay, see Additional file 1: Supplemental Table 2 for raw data) were below the LLoQ of the respective assay (determined as described above), and for statistical analysis, these samples were assigned values equal to the LLoQ of the assay. Statistical analysis was repeated with the actual readings of these 10 measurements, but produced similar results. The significance threshold was set to a two-sided *p* ≤ 0.05.

## Results

### Plasma Aβ42, NT1, and NfL levels are stable over a 5 day period and are not influenced by the time of day when blood is collected

**Fig. 1:**
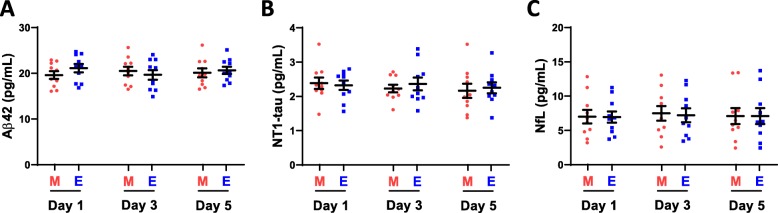
Plasma Aβ42, NT1 tau, and NfL levels are constant over a 5 day period. Plasma was collected from 10 healthy volunteers on three occasions at 2-day intervals (day 1, day 3, day 5). On each sample day, blood was collected at 2 time points: the first between 7 and 10 am (M), and the second between 4 and 7 pm (E). Specimens were analyzed using ultra-sensitive Simoa-based assays for **a** Aβ42, **b** NT1 tau, and **c** NfL. Shown are measurements for each of the analytes on day 1, day 3, and day 5. Each point represents a single measurement, and mean ± SEM are indicated. Values measured in morning samples (M) are in red circles, and evening specimens (E) are in blue squares. Mixed effect regression analysis revealed that plasma levels of **a** Aβ42, **b** NT1 tau, and **c** NfL were stable and not significantly influenced by time of day (morning vs. evening) and did not change over a 5-day interval (day 1 vs. day 3 vs. day 5)

The usefulness of any biomarker is, among other in vivo factors, influenced by whether it (i) remains constant over a reasonable period of time, (ii) is altered by diurnal factors, and (iii) requires pre-sampling fasting. Here, we sought to examine biomarkers of amyloid, tau, and neurodegeneration [29] in plasma of individuals with DS across the first seven decades of life. Before analyzing precious clinical samples, we conducted experiments to test the stability of analytes to be measured, namely Aβ42, NT1 tau, and NfL. Blood was collected from 10 healthy volunteers on days 1, 3, and 5. On each collection day, blood was obtained at two time points: (i) morning (after an overnight fasting) and (ii) evening. This design was employed to account for clinical practice in an outpatient setting in which patients are asked either to donate blood in the morning after overnight fasting, or to attend clinic later in the day without any dietary restrictions. Figure 1 shows the measured concentrations of Aβ42, NT1 tau, and NfL in the morning (red circles) and in the evening (blue squares). Spaghetti blots of individual measurements from all 10 healthy volunteers are presented in Supplemental Figures 1–3 (Additional file 2). Measurements for all three analytes in specimens collected in the morning and in the evening on three different days in 1 week were highly similar (Fig. 1). There was no statistically significant effect of time of day (morning vs. evening), and analyte levels were stable across the 5-day sampling period (day 1 vs. day 3 vs. day 5). The %CVs of measurements obtained on the morning of day 1, day 3, and day 5 in the same individual for Aβ42, NT1 tau, and NfL were 3.8% (range 0.9–6.2%), 9.3% (range 1.3–20.3%), and 7.3% (range 0.4–15.0%), respectively. Comparable %CVs were observed for measurements in the evening (Table 1). Collectively, these results demonstrate that the forms of Aβ, tau, and NfL in plasma measured by the Aβ42, NT1 tau, and NfL assay are stable, and do not appear to be influenced by diurnal and dietary factors. These findings have relevance both to the current study and for all studies measuring these analytes. Our study involved subjects from vulnerable populations (including individuals with DS, neonates, and young children) for whom it would be unreasonable to request overnight fasting for a research study, or to coordinate blood collection at a specific time of day. Thus, it is important that the measured analytes are not influenced by time of day or dietary intake.

**Table 1:** Biological variation of analytes

Assay	Daytime	Mean ± SD (range) level for all 10 donors (pg/mL)	Mean ± SD (range) intra-subject %CV for all 10 donors
Aβ42	Morning	20.07 ± 2.81 (16.1–26.2)	3.8 ± 1.9% (0.9–6.2%)
	Evening	20.47 ± 2.89 (16.3–25.1)	5.1 ± 3.4% (1.0–12.2%)
NT1 tau	Morning	2.31 ± 0.49 (1.4–3.4)	9.3 ± 5.3% (1.3–20.3%)
	Evening	2.26 ± 0.51 (1.5–3.5)	8.7 ± 7.3% (1.1–26.5%)
NfL	Morning	7.20 ± 3.30 (2.4–13.4)	7.3 ± 4.7% (0.4–15.0%)
	Evening	7.09 ± 3.03 (2.5–13.7)	8.9 ± 4.3% (3.3–17.1%)

*Abbreviations*: *SD* standard deviation, *%CV* percentage coefficient of variation

### Aβ1-42, NT1, and NfL plasma concentrations depend on age in Down syndrome individuals compared to controls

To study age-dependent changes of plasma AD biomarkers in DS, samples were obtained from 100 people with DS aged 3 months to 68 years. One hundred age- and sex-matched participants free of neurological disease served as controls (see Table 2 for participant characteristics).

**Table 2:** Demographics of DS and controls

			Down syndrome	Control
	Age groups (in years)	*N*	Age in years mean ± SD	Age in years mean ± SD
Mean age (year)	0–10	30	4.9 ± 2.9	5.0 ± 2.9
	11–20	23	16.2 ± 3.1	16.3 ± 3.2
	21–30	15	24.1 ± 2.8	24.0 ± 3.2
	31–40	10	35.7 ± 2.9	35.5 ± 3.0
	41–50	13	45.4 ± 3.6	45.2 ± 3.3
	> 50	9	57.1 ± 5.6	57.1 ± 5.6
	Age group (in years)	N	Number of females (% of total)	Number of females (% of total)
Sex (female)	0–10	30	15 (50.0)	15 (50.0)
	11–20	23	10 (43.5)	10 (43.5)
	21–30	15	4 (26.7)	4 (26.7)
	31–40	10	4 (40.0)	4 (40.0)
	41–50	13	4 (30.8)	4 (30.8)
	> 50	9	1 (11.1)	1 (11.1)

*Abbreviation*: *SD* standard deviation

**Fig. 2:**
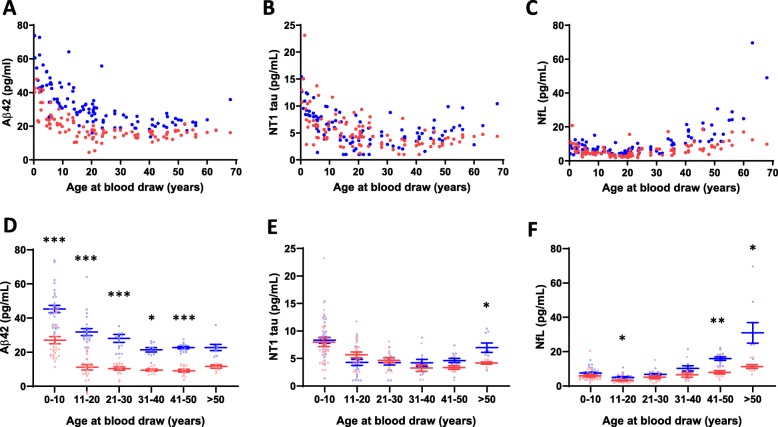
Plasma Aβ42, NT1 tau, and NfL change differentially with age in Down syndrome versus control subjects. Plasma samples from 100 individuals with DS and 100 controls matched for age and sex were analyzed with ultra-sensitive assays for **a**, **d** Aβ42, **b**, **e** NT1 tau, and **c**, **f** NfL. **a**–**c** Individual Aβ42, NT1 tau, and NfL values of subjects with DS (blue) and controls (red) are shown as a function of age. Each point represents one individual. **d**–**f** Data displayed in **a**–**c** are grouped in 10 year intervals. Each open circle represents a single individual. Mean and standard error of the mean are shown. **a**, **d** At any given age, the absolute amount of Aβ42 is higher in DS than in control subjects, but levels of Aβ42 decrease with age, with a greater decrease in DS. **b**, **e** NT1 tau levels tend to increase in older individuals with DS compared to controls. **c**, **f** NfL levels show a greater increase with age in individuals with DS than controls. Differences were assessed with paired *t* test (normally distributed data) or Wilcoxon signed-rank test (non-normally distributed data). *p* values were adjusted for multiple testing using Bonferroni correction. *p < 0.05; **p < 0.01; ***p < 0.001

**Fig. 3:**
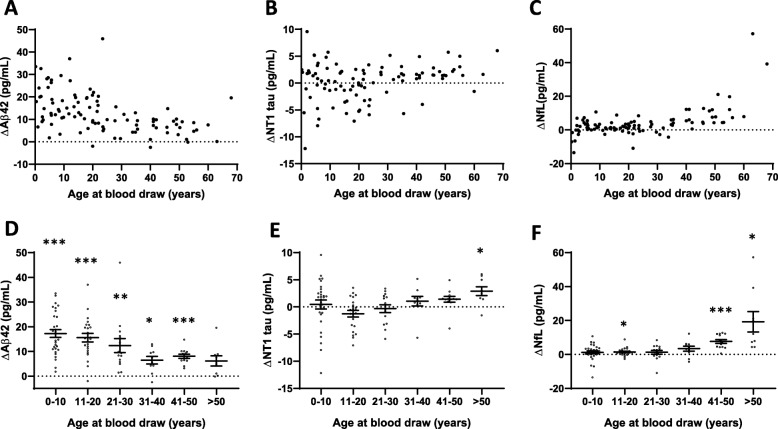
Relative levels of plasma Aβ42 decrease in DS with age, whereas the relative levels of NT1 tau and NfL tend to increase. To compare relative changes of plasma biomarkers in DS compared to controls, we calculated the difference between each sex- and age-matched pair (Δ = DS − control). For **a** ΔAβ42, **b** ΔNT1 tau, and **c** ΔNfL, each point represents a single difference value. **d**–**f** Difference values are also grouped in 10 year intervals. Each open circle represents a single individual. Mean and standard error of the group mean are shown. **a**, **d** Aβ42 decreases with age in DS. **b**, **e** NT1 tau is highly variable in the first 3 decades of life, but tends to increase in later life in DS. **c**, **f** ΔNfL is relatively constant in the first 30 years of life, but increases in DS with age. Differences were assessed with one sample *t* test (normally distributed data) or one sample Wilcoxon signed-rank test (non-normally distributed data). *p* values were adjusted for multiple testing using Bonferroni correction. *p < 0.05; **p < 0.01; ***p < 0.001

Individual results for all 3 analytes are presented in Supplemental Table 2 (Additional file 1). Concentrations of Aβ42 were higher in individuals with DS than controls across the entire age range (Fig. 2 a, d and Table 3). In the youngest age group (0–10 years), mean Aβ42 concentrations were approximately 1.6-fold higher in DS individuals compared to controls (45.3 vs. 28.1 pg/mL, *p* < 0.001), but the differences between plasma Aβ1-42 levels in individuals with DS and controls declined with increasing age (Fig. 3a, d). At approximately 30 years of age, Aβ42 levels reached a steady level of ~ 22 pg/mL for individuals with DS and ~ 15 pg/mL for controls. In the oldest age group (> 50 years), mean Aβ42 concentrations were ~ 1.4-fold higher in individuals with DS compared to controls (22.7 pg/mL vs. 16.6 pg/mL, *p* = 0.07).

**Table 3:** Biomarkers stratified by the 10-year age group

Analyte	Age group (in years)	Down syndrome	Control	*p* value*
Aβ42 (pg/mL)	0–10	45.3 ± 2.1	28.1 ± 1.6	< 0.001
	11–20	31.8 ± 2.0	16.2 ± 1.2	< 0.001
	21–30	28.1 ± 2.3	15.7 ± 0.8	< 0.001
	31–40	21.4 ± 1.3	15.0 ± 1.5	0.014
	41–50	22.7 ± 0.7	14.7 ± 0.6	< 0.001
	> 50	22.7 ± 1.8	16.6 ± 0.8	n.s.
NT1 tau (pg/mL)	0–10	8.3 ± 0.5	7.9 ± 0.8	n.s.
	11–20	4.3 ± 0.5	5.6 ± 0.5	n.s.
	21–30	4.3 ± 0.4	4.6 ± 0.4	n.s.
	31–40	4.2 ± 0.6	3.2 ± 0.6	n.s.
	41–50	4.6 ± 0.4	3.2 ± 0.3	n.s.
	> 50	7.0 ± 0.8	4.1 ± 0.2	0.042
NfL (pg/mL)	0–10	7.5 ± 0.5	6.3 ± 0.6	n.s.
	11–20	5.1 ± 0.4	3.6 ± 0.3	0.010
	21–30	6.7 ± 0.4	5.4 ± 0.9	n.s.
	31–40	10.2 ± 1.6	6.8 ± 1.3	n.s.
	41–50	15.9 ± 1.0	8.3 ± 1.0	0.001
	> 50	31.0 ± 6.0	11.7 ± 1.1	0.023

Mean concentrations ± SEM

*p value adjusted for multiple comparisons in age strata by *Bonferroni* correction

For NT1 tau, levels were highest in children and fell with age in both the DS and control groups (Fig. 2b, e and Table 3). In general, the levels of NT1 tau in DS and controls broadly overlapped, but tended to diverge after the age of 30 (Fig. 3b, e and Table 3). Specifically, in individuals with DS 50 years and older, NT1 levels were significantly increased compared to controls (7.0 vs. 4.0 pg/mL, *p* = 0.042) (Fig. 3e, Table 3).

For the first three decades of life, plasma NfL levels were relatively constant and were similar in DS and controls (Fig. 2c, f and Fig. 3c, f, Table 3). In subjects over 30 years of age, NfL levels increased in both controls and DS, but the increase was considerably greater for people with DS. Specifically, NfL levels were significantly increased in individuals with DS compared to controls at ages 40 and older (41–50 years, 15.9 vs. 8.3 pg/mL, *p* = 0.001; > 50 years, 31.0 vs. 11.7, *p* = 0.023) (Fig. 2f, Table 3).

We also applied more sophisticated statistical models in an attempt to better understand differences in the age-dependent changes in DS versus controls. Specifically, we used piecewise linear regression to determine time points of slope changes in DS and controls (Additional file 1: Supplemental Table 3 and Additional file 2: Supplemental Figure 4). Minimum levels of Aβ42 in DS were reached at 26 years, whereas in controls, the minimum occurred at 15 years. In DS, the infliction point for NT1 was 17 years, and in controls 29 years, and only in old age was NT1 higher in DS individuals than controls. NfL levels increased with age in both DS and control individuals, but the slope was much steeper in DS compared to controls (1.05 pg/mL vs. 0.19 pg/mL per year). While these analyses suggest some important trends, it is important to note that the wide confidence intervals associated with estimates for time points of slope change make it problematic to assign reliable change-points.

## Discussion

Here, we analyzed changes in biomarkers related to 3 primary features of AD and AD in DS: amyloid, tau, and neurodegeneration [29]. In the absence of large longitudinal cohorts, we gathered specimens from 100 DS individuals aged from 3 months to 68 years and compared their values with those of age- and sex-matched controls. We found that Aβ42 levels were higher in DS than in controls, regardless of age. In both DS and controls, Aβ42 levels were highest in neonates. NT1 tau levels were similar in DS and controls across all ages, except for older ages when NT1 tau was higher in DS than controls. For both DS and controls, NfL levels were relatively low in age groups up to ~ 30 years, whereas in older age groups, NfL was higher and the increase was greater in DS than in controls.

As expected for individuals with 3 copies of the *APP* gene (and consistent with prior studies [17, 30–32]), we observed higher plasma Aβ42 levels in individuals with DS compared to controls. In the first decade of life, a time when there is little amyloid deposition [33, 34], plasma Aβ42 levels were on average a little higher (~ 1.6 fold) in DS than the expected 1.5-fold elevation due to gene dosage (*p* = 0.03). Why this should be is unclear, but it is worth noting that several AD risk factors are encoded on chromosome 21 [2] and these might contribute to either enhanced amyloidogenic processing of APP [35, 36] or reduced degradation of Aβ [37]. Also, neuronal Aβ production is activity dependent [38], and in DS, there is evidence of aberrant hyperactivity during development and early life [39] that could contribute to higher Aβ levels.

Aβ42 levels tended to fall with age in both individuals with DS and controls, but the relative decrease was greater in DS. There was a strong tendency for the DS/control Aβ42 ratio to be lower in the oldest group (> 50 years) compared to youngest group (0–10 years); however, this did not reach statistical significance (1.6 vs. 1.4, *p* = 0.08). Nonetheless, the trend is consistent with the notion that Aβ42 is prone to aggregate and becomes trapped in accumulating plaques and is in line with multiple AD studies linking falling CSF and plasma Aβ42 with increased cerebral amyloid deposition [40, 41]. However, prior studies examining the association of plasma Aβ42 with age in DS have yielded conflicting results with reports of increased [42], decreased [43], and unchanged Aβ42 levels [14, 31, 44]. But previous studies were not designed to look at the effect of age across a broad age span. We found that plasma Aβ42 levels fell sharply in the first 3 decades of life in individuals with DS, but were relatively stable in the age range from 31 to 68 years. Our results from a broad age range of individuals (3 months to 68 years) provide the perspective to better understand what had formerly appeared discordant results, that is, decreasing Aβ42 levels in younger DS individuals [43] but relatively stable levels in older DS individuals [14, 31, 44].

Human plasma is a complex matrix, components of which can interfere with immunoassays. One means of overcoming matrix interference is to dilute samples so as to reduce interfering plasma components to a level below which they no longer interfere. This requires that the assays employed are sufficiently sensitive to allow dilutions necessary to preclude matrix interference. Here, we employed ultra-sensitive assays and evaluated the maximum dilution that allowed consistent detection of analytes across a large number of human samples. For Aβ42, only a few studies [27, 32, 45, 46] have used such ultra-sensitive techniques.

In contrast to Aβ, only a handful of studies have attempted to measure tau in plasma of DS individuals. Extracellular tau is molecularly complex [20–22], and different assays detect distinct populations of tau alloforms, complicating comparisons of results obtained using different assays. Here, we utilized our in-house NT1 tau assay which we have previously shown to be capable of detecting forms of tau that are significantly elevated in plasma of patients with AD-MCI and mild AD [21]. Like Aβ42, NT1 detected tau was highest at early age (0–10 years). NT1 levels fell between 11 and 30 years but thereafter increased. This pattern is a mirror image of the Aβ42 results, with both exhibiting pivotal changes between 20 and 40 years.

NfL, a now widely validated marker of neurodegeneration [18], was relatively low in early life, but in both controls and DS, NfL increased steadily after age 30. Importantly, NfL levels in plasma of individuals with DS started to diverge from control levels in the 31–40-year age group and were most different in the two oldest age groups.

Collectively, our findings demonstrate that plasma measures of amyloid, tau, and neurodegeneration change with age and that the relative differences in these markers are greatest in the 31–40-, 41–50-, and over 50-year age groups. In our study cohort, elevated concentrations of NfL and NT1 measured tau in older individuals with DS are consistent with the following: (i) recent cross-sectional studies that found increased plasma NfL and tau in prodromal and AD dementia in people with DS [32, 47], and (ii) an increasing prevalence of AD in older individuals with DS [4, 5]. Notwithstanding the identification of these important trends, our results indicate that it will be impractical to use a single time point measurement of these biomarkers to diagnose AD in DS. Rather, our data support longitudinal assessment of these markers to further evaluate their potential to predict onset of disease.

A particular strength of this study is the use of a relatively large number of DS individuals (*n* = 100) and age- and sex-matched controls (*n* = 100) with a broad age range (3 months to 68 years). Another strength is the use of analytically validated methods and testing conditions. The major weaknesses of our study include the fact that the study is cross-sectional and not longitudinal, the use of controls from a biobank, and that we did not include cognitive assessments. Future studies should collect clinical information, such as cognitive measures, APOE status, and concomitant medication, and it may be useful to measure other alloforms of Aβ so as to calculate ratios of different Aβ species (e.g., Aβ42/40, Aβ42/38).

## Conclusions

Viewed together, the trajectories of all three biomarkers point towards important changes after the third decade of life in people with DS. During that period, Aβ42 in DS falls considerably and NT1 and NfL levels rise steadily. In order to identify individuals with DS who would most benefit from secondary prevention treatments, future prospective longitudinal studies should focus on the critical age span between 30 and 50 years measuring both biomarker changes and cognition. Our successful use of sensitive and dynamic plasma analytes reflecting the cardinal features of AD is particularly salient for people with DS, given the ethical challenges of trying to use CSF or brain imaging to monitor their temporal progression into AD.

## Supplementary information

**Additional file 1:Table S1.** Demographics of the PRECISION study cohort. **Table S2.** Individual results of plasma Aβ42, NT1 tau, and NfL in Down syndrome and controls. **Table S3.** Change-point regression for association of age with plasma biomarker levels.

**Additional file 2:Figure S1.** Plasma Aβ42 levels are stable when measured in the same subjects over a 5 day period. **Figure S2.** Plasma NT1 tau levels are stable in the same subjects over a 5 day period. **Figure S3.** Plasma NfL levels are stable in the same subjects over a 5 day period. **Figure S4.** Prediction of biomarker changes by age using piecewise linear regression.

## Data Availability

Raw data of plasma measurements in DS and controls are reported in Supplemental Table 2 (Additional file 1). All other data supporting the conclusions of this manuscript will be made available by the corresponding authors, without undue reservation, to any qualified researcher. All materials used in the publication are commercially available.

## Abbreviations

Aβ42: Amyloid β-protein ending at amino acid 42
AD: Alzheimer’s disease
APOE: Apolipoprotein E
APP: Amyloid precursor protein
CSF: Cerebrospinal fluid
DS: Down syndrome
EDTA: Ethylenediaminetetraacetic acid
LLoQ: Lower limit of quantitation
NT1 tau: N-terminal tau 1 assay (detects forms of tau containing at least residues 6–198)
NfL: Neurofilament light
PET: Positron emission tomography
Simoa: Single molecule array
%CV: Percent coefficient of variation
