# Supplementary material for: Dynamics of plasma biomarkers in Down syndrome: the relative levels of Aβ42 decrease with age, whereas NT1 tau and NfL increase
Source: Alzheimers Res Ther. 2020 Mar 19;12:27. doi: 10.1186/s13195-020-00593-7 (PMC7081580; doi:10.1186/s13195-020-00593-7)
Supplement: Supplementary file 1 — Additional file 1:Table S1. Demographics of the PRECISION study cohort. Table S2. Individual results of plasma Aβ42, NT1 tau, and NfL in Down syndrome and controls. Table S3. Change-point regression for association of age with plasma biomarker levels. [file 13195_2020_593_MOESM1_ESM.docx]

**Supplemental Table 1: Demographics of the PRECISION study cohort**

| **N** | **Mean age ± SD** | **Median age (range)** | **Male (%)** |
| --- | --- | --- | --- |
| 10 | 44.1 ± 9.6 years | 43 years (30-53 years) | 8 (80%) |

**Supplemental Table 2: Individual results of plasma Aβ42, NT1 tau, and NfL in Down syndrome and controls.**

| **Diagnostic Group** | **Age (in years)** | **Aβ42 (pg/mL)** | **NT1 tau (pg/mL)** | **NfL (pg/mL)** |
| --- | --- | --- | --- | --- |
| Down syndrome | 0.2  0.5  1.0  1.4  1.7  2.0  2.1  2.4  2.5  2.8  3.1  4.1  4.5  4.6  5.0  5.5  5.6  5.7  5.7  5.7  5.8  6.1  7.1  8.1  8.4  8.7  9.0  9.3  9.5  9.9  11  12  12  12  14  14  14  14  15  15  16  16  17  18  18  18  19  19  20  20  20  20  20  21  22  22  22  22  22  23  24  24  24  25  28  28  28  30  31  32  34  35  35  35  36  36  40  40  41  41  41  42  42  46  46  46  48  49  49  50  50  51  53  53  55  55  56  60  63  68 | 73.8  60.6  54.6  47.3  42.6  72.8  62.3  43.0  40.8  43.8  56.4  52.3  44.4  52.6  25.5  34.3  45.2  45.9  34.4  48.7  45.0  42.4  36.1  36.1  34.4  33.6  45.8  31.9  43.2  30.8  31.8  36.3  64.2  45.0  40.7  34.3  42.9  30.1  28.2  26.9  20.4  28.6  30.0  32.7  33.1  22.7  30.1  28.0  33.2  16.0  26.3  22.7  27.5  28.5  35.2  31.1  29.7  30.8  23.8  55.8  22.4  19.0  23.5  26.2  19.1  27.4  29.0  19.1  26.7  25.3  21.0  18.9  25.9  22.7  22.6  18.9  18.2  13.7  24.1  20.8  24.2  19.9  21.7  20.2  19.3  27.7  25.7  24.6  22.3  20.8  23.8  23.6  21.3  17.4  22.5  22.1  20.4  23.8  17.6  35.9 | 15.4  12.7  9.5  10.9  8.4  12.4  9.2  12.3  8.4  9.0  8.2  8.7  8.0  11.5  8.1  6.2  7.4  1.4  7.3  5.8  7.8  5.3  8.0  4.6  3.9  10.0  6.6  9.9  6.7  5.7  7.0  6.0  7.3  9.3  8.8  3.7  4.5  7.1  0.0*  4.3  0.8*  3.7  3.1  4.1  3.3  1.7  8.3  2.1  2.8  0.3*  3.6  1.1  4.3  4.4  5.1  5.0  7.0  0.1*  5.4  5.9  3.8  0.3*  2.9  4.4  5.2  4.5  5.2  2.9  8.8  2.6  2.3  5.6  4.1  4.0  4.3  2.0  3.4  5.0  5.9  4.3  3.9  4.9  1.6  4.0  5.9  5.1  7.4  3.6  3.9  4.5  5.3  9.9  5.5  6.0  6.4  9.7  5.6  2.8  6.4  10.4 | 4.2  8.9  7.3  3.6  7.6  8.0  12.4  4.9  7.2  5.0  10.5  9.6  6.6  12.4  7.6  8.9  7.7  11.1  5.0  5.7  7.1  6.7  4.2  5.7  7.0  15.1  4.8  6.5  6.7  8.2  4.3  5.4  7.9  5.9  5.6  5.6  4.4  6.5  4.3  3.6  3.4  10.7  4.3  2.3  4.6  4.2  5.9  4.1  6.4  4.3  4.8  2.9  5.1  7.0  4.7  4.1  5.5  8.0  7.0  10.3  7.3  6.1  4.7  7.0  6.6  8.2  8.4  5.7  4.2  4.5  13.0  12.2  10.5  7.1  9.7  9.4  10.3  21.4  17.5  14.5  18.3  13.6  11.7  11.5  22.3  18.7  18.4  21.1  11.4  12.5  15.8  30.7  11.5  15.7  24.0  28.9  24.3  24.9  69.6  49.1 |
| Controls | 0.3  0.4  1.0  1.4  1.7  2.0  2.0  2.4  2.5  3.0  3.2  4.4  4.5  4.5  5.3  5.4  5.6  5.6  5.8  5.8  6.1  6.2  7.1  8.1  8.2  8.7  9.0  9.1  9.6  9.8  11  12  12  12  14  14  14  14  15  15  16  16  17  18  18  19  19  19  20  20  20  21  21  21  21  21  21  22  22  24  24  24  24  25  28  28  28  30  31  32  34  35  35  36  36  36  40  40  41  41  41  42  43  45  46  46  46  48  49  49  50  51  52  53  55  56  56  60  63  68 | 40.3  42.6  47.9  23.3  22.6  40.2  42.1  34.1  29.7  34.0  41.6  24.2  20.2  24.0  23.7  21.6  18.8  21.2  34.0  26.6  26.1  25.1  24.1  21.8  20.8  30.2  16.3  21.2  24.9  19.8  16.7  23.8  27.2  17.8  25.1  23.2  22.1  10.4  10.6  13.9  16.3  16.5  19.6  13.4  13.4  16.8  14.8  4.5  12.4  18.0  17.6  13.3  5.4  20.3  17.2  17.6  13.4  10.6  15.5  9.8  15.7  14.0  17.8  16.3  17.5  18.1  13.4  17.7  14.5  15.7  16.7  14.6  13.2  13.0  15.2  13.2  17.2  16.2  15.0  10.6  16.9  13.7  11.9  13.7  15.2  12.9  16.5  12.2  16.9  17.6  17.4  14.7  21.4  17.4  17.8  22.1  20.4  23.8  17.6  35.9 | 12.9  10.7  15.1  23.1  9.5  2.9  7.4  10.4  6.1  7.1  10.9  7.3  2.8  6.4  6.4  6.6  4.2  6.1  6.5  13.7  4.1  12.0  7.6  5.6  4.0  5.2  3.9  4.1  6.3  7.0  11.6  5.7  8.5  5.8  7.0  4.2  2.5  4.9  2.3  7.8  6.6  7.0  4.3  4.7  2.5  8.8  5.7  4.3  2.8  6.3  4.0  1.2  9.4  4.9  4.4  2.6  5.3  4.3  6.3  2.5  8.0  6.9  6.0  4.3  3.2  2.9  2.3  4.6  3.6  1.9  1.1  3.8  1.0  3.6  2.8  7.7  2.6  3.4  0.7*  2.9  3.0  3.0  5.5  2.5  3.1  3.7  5.2  2.5  2.7  3.1  3.9  4.1  3.0  3.9  3.3  4.7  4.2  4.4  4.7  4.4 | 11.3  10.5  20.9  10.1  7.8  5.3  5.5  5.0  9.9  5.9  8.4  3.8  2.3  5.1  4.1  7.5  4.0  5.5  5.6  4.3  4.4  5.9  4.2  5.2  3.9  4.4  3.5  4.6  5.1  4.8  4.5  8.0  6.6  5.2  4.6  4.2  3.7  2.2  3.0  3.7  2.9  1.2*  3.2  3.0  2.4  2.3  2.5  2.8  2.8  3.3  1.8*  4.5  2.6  2.4  15.7  6.4  4.5  3.0  4.4  1.8*  6.5  3.4  5.5  7.4  4.8  3.3  5.0  7.0  7.0  3.4  17.2  6.2  3.1  7.3  4.3  5.6  4.8  9.2  5.2  8.5  6.0  9.2  11.2  6.8  18.4  5.9  7.1  9.1  3.8  8.2  8.0  9.6  7.1  11.9  9.2  17.0  11.3  17.0  12.4  9.9 |

***measured values below the LLoQ of the assay**

**Supplemental Table 3: Change-point regression for association of age with plasma biomarker levels**

| **Analyte** | **Parameter** | **Coefficient** | **SE** | ***p*-value** | **95% CI** |
| --- | --- | --- | --- | --- | --- |
| Aβ42 in DS  Aβ42 in CTL | Change-point  Left slope  Right slope  Constant  Change-point  Left slope  Right slope  Constant | 25.71  -1.16  0.03  51.79  15.29  -1.39  0.04  35.60 | 2.78  0.13  0.13  1.85  1.39  0.17  0.04  1.41 | <0.001  <0.001  0.801  <0.001  <0.001  <0.001  0.407  <0.001 | 20.19 to 31.23  -1.42 to -0.90  -0.22 to 0.29  48.11 to 55.47  12.54 to 18.05  -1.73 to -1.04  -0.05 to 0.12  32.81 to 38.39 |
| NT1 in DS  NT1 in CTL | Change-point  Left slope  Right slope  Constant  Change-point  Left slope  Right slope  Constant | 16.76  -0.46  0.07  10.80  28.70  -0.22  0.04  9.18 | 1.69  0.07  0.02  0.63  4.68  0.04  0.05  0.62 | <0.001  <0.001  0.001  <0.001  <0.001  <0.001  0.471  <0.001 | 13.41 to 20.11  -0.60 to -0.31  0.03 to 0.12  9.55 to 12.05  19.41 to 37.98  -0.30 to -0.14  -0.06 to 0.14  7.95 to 10.41 |
| NfL in DS  NfL in CTL | Change-point  Left slope  Right slope  Constant  Change-point  Left slope  Right slope  Constant | 32.74  -0.08  1.05  7.50  14.52  -0.40  0.19  8.66 | 2.05  0.07  0.11  1.14  2.16  0.12  0.03  0.94 | <0.001  0.279  <0.001  <0.001  <0.001  0.001  <0.001  <0.001 | 28.67 to 36.80  -0.22 to 0.06  0.83 to 1.26  5.24 to 9.77  10.24 to 18.81  -0.65 to -0.16  0.13 to 0.25  6.80 to 10.53 |
