# Supplementary figures and images for: Dynamics of plasma biomarkers in Down syndrome: the relative levels of Aβ42 decrease with age, whereas NT1 tau and NfL increase
Source: Alzheimers Res Ther. 2020 Mar 19;12:27. doi: 10.1186/s13195-020-00593-7 (PMC7081580; doi:10.1186/s13195-020-00593-7)

# Supplemental Figure 1

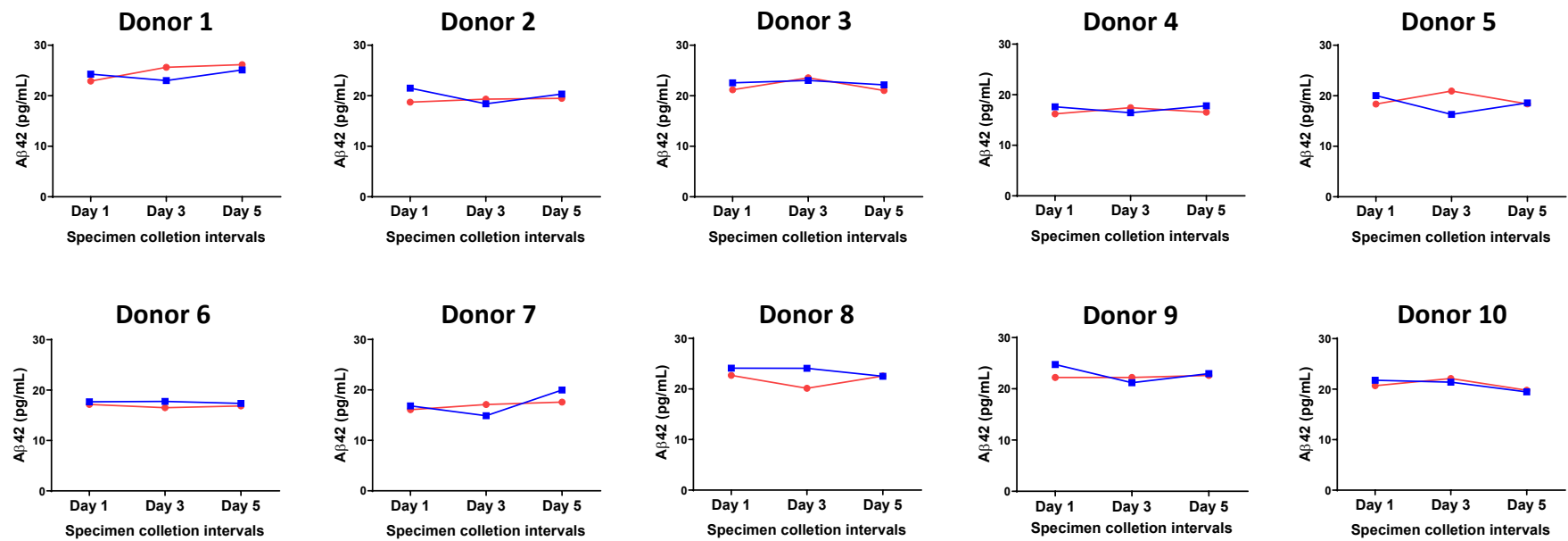

# Supplemental Figure 2

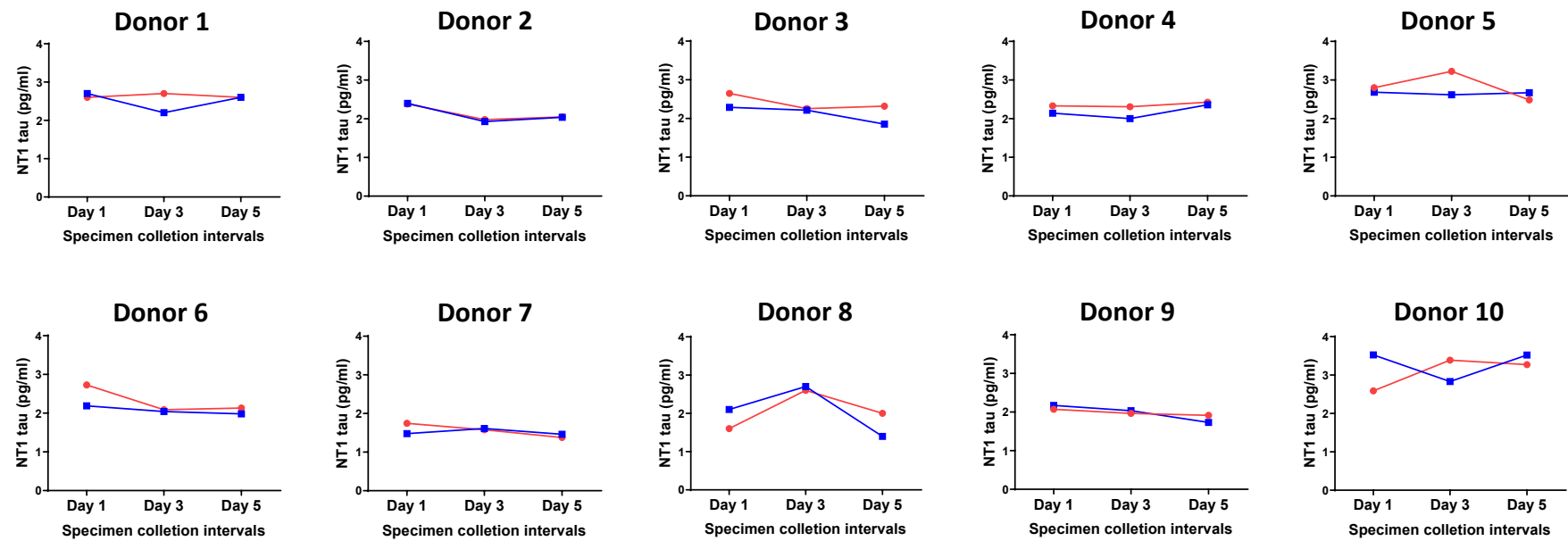

# Supplemental Figure 3

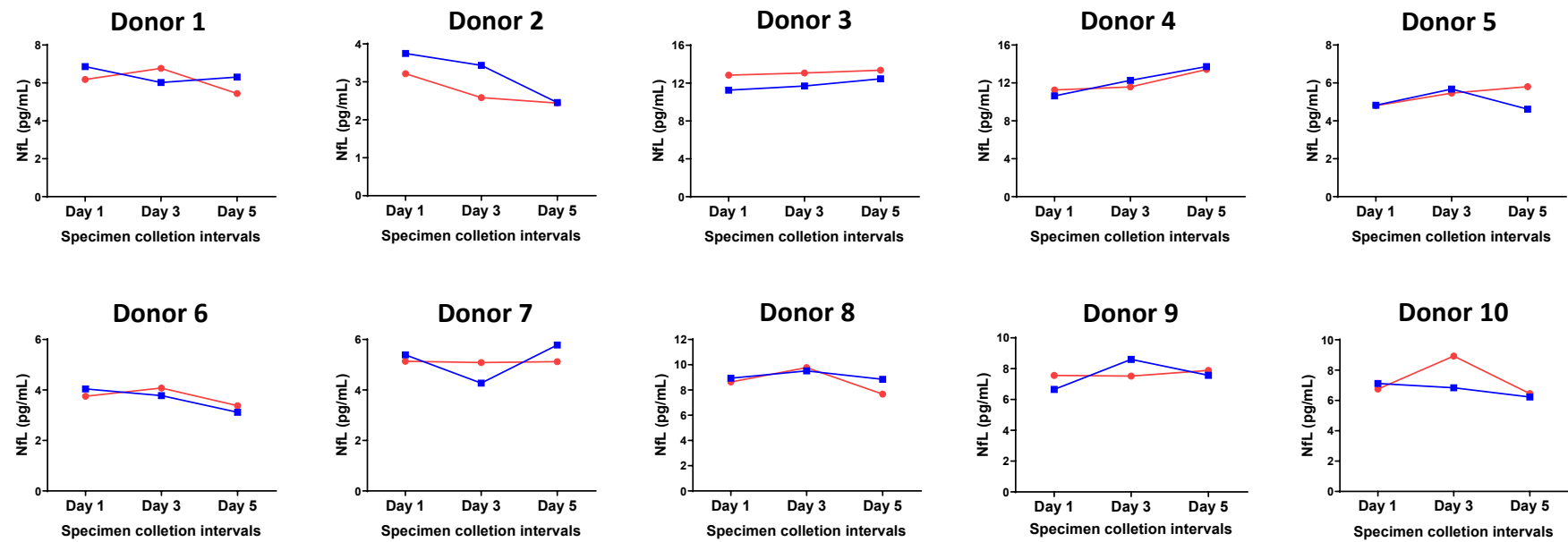

Supplemental Figure 4

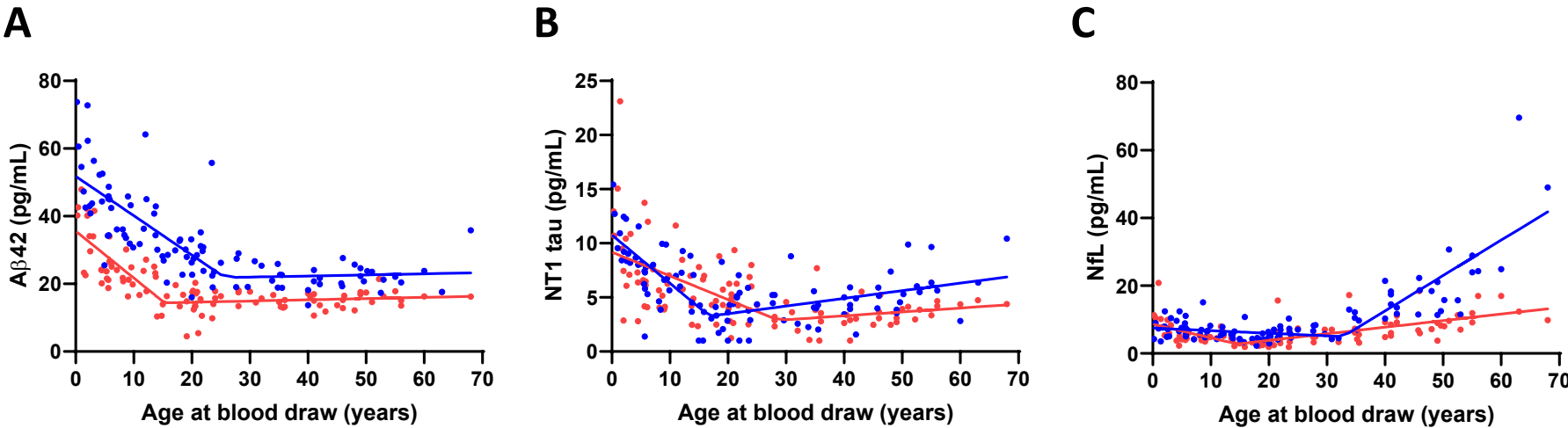

Supplement: Supplementary file 2 — Additional file 2:Figure S1. Plasma Aβ42 levels are stable when measured in the same subjects over a 5 day period. Figure S2. Plasma NT1 tau levels are stable in the same subjects over a 5 day period. Figure S3. Plasma NfL levels are stable in the same subjects over a 5 day period. Figure S4. Prediction of biomarker changes by age using piecewise linear regression. [file 13195_2020_593_MOESM2_ESM.pdf]
